# Fructose-Induced Carbonyl/Oxidative Stress in *S. cerevisiae*: Involvement of TOR

**DOI:** 10.1155/2016/8917270

**Published:** 2016-02-25

**Authors:** Bohdana V. Valishkevych, Ruslana A. Vasylkovska, Liudmyla M. Lozinska, Halyna M. Semchyshyn

**Affiliations:** ^1^Department of Biochemistry and Biotechnology, Vasyl Stefanyk Precarpathian National University, 57 Shevchenko Street, Ivano-Frankivsk 76018, Ukraine; ^2^Department of Biology, Lund University, Sölvegatan 35, 223 62 Lund, Sweden

## Abstract

The TOR (target of rapamycin) signaling pathway first described in the budding yeast* Saccharomyces cerevisiae* is highly conserved in eukaryotes effector of cell growth, longevity, and stress response. TOR activation by nitrogen sources, in particular amino acids, is well studied; however its interplay with carbohydrates and carbonyl stress is poorly investigated. Fructose is a more potent glycoxidation agent capable of producing greater amounts of reactive carbonyl (RCS) and oxygen species (ROS) than glucose. The increased RCS/ROS production, as a result of glycoxidation* in vivo*, is supposed to be involved in carbonyl/oxidative stress, metabolic disorders, and lifespan shortening of eukaryotes. In this work we aim to expand our understanding of how TOR is involved in carbonyl/oxidative stress caused by reducing monosaccharides. It was found that in fructose-grown compared with glucose-grown cells the level of carbonyl/oxidative stress markers was higher. The defects in the TOR pathway inhibited metabolic rate and suppressed generation of glycoxidation products in fructose-grown yeast.

## 1. Introduction

A strong positive correlation between the intake of excessive dietary carbohydrates and metabolic disorders has been observed in many experimental and clinical studies [1–6]. Among the mechanisms thought to be responsible for metabolic disturbances, increased ROS/RCS production, as a result of glycoxidation, is the most well supported one [1, 3]. Glucose is the least reactive reducing monosaccharide, and this characteristic is considered to be responsible for the emergence of glucose as the primary metabolic fuel [7–9]. At the same time, fructose, the intake of which increased considerably during the past several decades [4, 6], is suggested to be more extensively than glucose involved in nonenzymatic processes and generation of reactive species [10–12]. The detrimental effects of long-term application of fructose in different experimental models can be explained by high level of glycoxidation products [4, 13, 14]. For instance, the excessive fructose intake in animals has been demonstrated to cause carbonyl/oxidative stress [10, 12].

It is well documented that signaling through the TOR pathway is activated by various extracellular and intracellular challenges when conditions are favorable for growth [15]. For example, TOR promotes cell growth in response to nutrient availability [16, 17]. Most studies on TOR activation by nutrients were focused on nitrogen sources [15, 18, 19], while carbohydrates have received less attention. In addition, TOR, as a central controller of cell growth, may respond to different types of stress and plays an important role under stressful conditions other than nutrient limitation [18]. However, little is known regarding relationship between TOR and carbonyl stress.

Here, using fructose- and glucose-grown yeast as a model, we have demonstrated that deletion of the TOR genes inhibited metabolic rate and suppressed generation of glycoxidation products in cells cultivated on fructose but not glucose.

## 2. Material and Methods

### 2.1. Yeast Strains and Chemicals

The* Saccharomyces cerevisiae* strains were used as follows: YPH250 (wild type* MAT* **a** *trp1-Δ1 his3-Δ200 lys2-801 leu2-Δ1 ade2-101 ura3-52*), kindly provided by Professor Yoshiharu Inoue (Kyoto University, Japan) [20]; JK9-3da (wild type* MATa leu2–3,112 ura3–52 rme1 trp1 his4 GAL+ HMLa*) [21] and its derivatives MH349-3d (JK9-3da,* tor1::LEU2-4*) [22], SH121 (JK9-3da,* tor2::ADE2-3/YCplac111::tor2-21ts*) [23], and SH221 (JK9-3da,* tor1::HIS3-3 tor2::ADE2-3/YCplac111::tor2-21ts*) [24], kindly provided by Professor Michael Hall (University of Basel, Switzerland). Chemicals were obtained from Sigma-Aldrich Chemical Co. (USA) and Fluka (Germany). All chemicals were of analytical grade.

### 2.2. Growth Conditions and Cell Extracts

Yeast cells were grown at 28°С with shaking at 175 r.p.m. in a liquid medium containing 1% yeast extract, 2% peptone, and 1% sucrose (YPS) for 24 h. The obtained culture was split into three groups: one diluted in YPS, another diluted in a medium containing 1% yeast extract, 2% peptone, and 2% glucose (YPD), and the last one diluted in a medium containing 1% yeast extract, 2% peptone, and 2% fructose (YPF). In all diluted cultures (~0.3 × 10^6^ cells/mL) cells were grown under the conditions mentioned above for additional 24 h. Cells from experimental cultures were collected by centrifugation (5 min, 8000 g) and washed with 50 mM potassium phosphate buffer (pH 7.0). The yeast cells from the first group were incubated at 28°С with: (1) 10–60% glucose- or 10–60% fructose-containing buffer solution for 24 h [25]; (2) 10 mM H_2_O_2_ for 1 h [26]; and (3) 50 mM glyoxal for 1 h [27].

The yeast pellets from respective experimental cultures were resuspended in lysis buffer (50 mM potassium phosphate buffer, 1 mM phenylmethylsulphonyl chloride, and 0.5 mM EDTA). Cell extracts were prepared by vortexing yeast suspensions with glass beads (0.5 mm) as described earlier [28] and kept on ice for immediate use.

### 2.3. Evaluation of Yeast Viability and Metabolic Activity

Cell viability was used as a measure of cell survival and was assessed by dilution spots visualization as a qualitative-comparative method [29]. Spot assays were performed by inoculating yeast cells (at the same concentration within each set) and their serial dilutions (1, 10^−2^, and 10^−4^) as a single drop of 5 *μ*L on an agar plate. After 3 days of incubation at 28°С, the resulting colonies formed clearly visible culture spots.

To evaluate metabolic activity of yeast 2,3,5-triphenyl tetrazolium chloride was used. Metabolically active cells are capable of reducing the dye to a water-insoluble red formazan that can be extracted from the cells with ethanol/acetone mixture, and the absorbance of this solution was then read at 485 nm [30]. The results are expressed as OD_485_ units per 10^8^ cells.

### 2.4. Fluorescent Assay of ROS

The fluorescent, oxidation-sensitive probe 2′,7′-dichlorofluorescein diacetate was used to measure the level of intracellular ROS [26]. The intensity of fluorescence of oxidized dichlorofluorescein diacetate was determined using *λ*_excitation_ = 500 nm and *λ*_emission_ = 520 nm with a SpectraMAX Gemini EM 96-well plate spectrofluorometer and Soft Max Pro 4.7 software (both from Molecular Devices, Sunnyvale, CA). The results are expressed in relative fluorescence units per 10^6^ cells.

### 2.5. Assay of Protein Carbonyls, Glycated Proteins, and *α*-Dicarbonyl Compounds

The parameters were measured spectrophotometrically with a Spekol 211 spectrophotometer (Carl Zeiss, Germany) and СФ-46 (ЛОМО, USSR). The content of carbonyl groups in proteins was measured by determining the amounts of 2,4-dinitrophenylhydrazone formed under reaction with 2,4-dinitrophenylhydrazine [28]. Carbonyl content was calculated from the absorbance maximum of 2,4-dinitrophenylhydrazone measured at 370 nm using an extinction coefficient of 22 mM^−1^·cm^−1^. The results are expressed in nmoles per mg of protein.

The content of glycated proteins was estimated by the fructosamine assay based on the reduction of nitroblue tetrazolium (NBT) at alkaline pH [31]. Cellular homogenates were diluted to a protein concentration of 1 mg/mL and dialyzed against water for 20 h to remove low-molecular-weight compounds. NBT in 100 mM carbonate buffer (pH 10.3) was added to a sample containing 0.2 mg protein to obtain a final NBT concentration of 0.3 mM. The absorbance was measured at 525 nm after 5 h incubation at 37°С. Content of glycated proteins was calculated using an extinction coefficient of 12.6 mM^−1^·cm^−1^. The results are expressed in nmoles per mg of protein.

*α*-Dicarbonyl compounds were measured by the Girard-T reaction [32]. The absorbance of the disubstituted compound, which is formed by binding of two Girard-T reagent molecules to dicarbonyl groups, was measured at a pH of 9.2 and a maximum absorption wavelength of 325 nm using an extinction coefficient of 18.8 mM^−1^·cm^−1^ for glyoxal. The results are expressed in nmoles of glyoxal equivalents per mg of protein.

### 2.6. Protein Concentration Measurement and Statistical Analysis

Protein concentration was determined by the Coomassie brilliant blue G-250 dye-binding method [33] with bovine serum albumin as the standard. Experimental data are expressed as the mean value of 3–7 independent experiments ± the standard error of the mean (SEM), and statistical testing used Student's *t*-test.

## 3. Results and Discussion

Fructose is commonly used as a sweetener and its intake has quadrupled since the early 1900s [4, 6]. This parallels the increase in obesity, diabetes mellitus, and other metabolic disorders [3, 5, 34–36]. The enhanced level of glycoxidation products is suggested to be involved in fructose negative effect in different experimental models [4, 13, 14, 36].

To compare glucose and fructose involvement in ROS/RCS generation, recently we used baker's yeast as* in vitro* and* in vivo* model and found that* in vitro* fructose compared to glucose was a more potent initiator of the glycoxidation reactions [25]. However, the vital fructose effects depended very much on the experimental conditions: (i) higher level of carbonyl/oxidative stress markers correlated with a higher aging rate of fructose-grown compared with glucose-grown yeast at the stationary phase (detrimental effect of fructose in long-term model) [11]; (ii) fructose-grown yeast at the exponential phase exposed to H_2_O_2_ demonstrated higher survival and lower intracellular concentration of ROS than glucose-grown cells (defensive effect of fructose in short-term model) [26]; and (iii) no difference between the glucose and fructose effects on the yeast survival and intracellular level of glycoxidation products under monosaccharide-induced stress, when intact cells were exposed to high concentrations of the hexoses [25].

**Figure 1:**
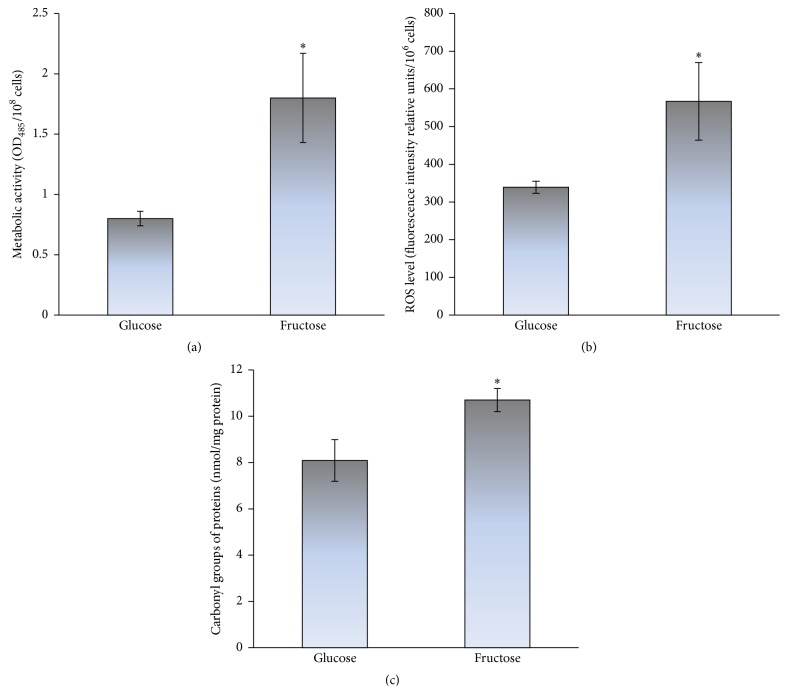
The metabolic rate (a) and level of oxidative stress markers: ROS (b) and protein carbonyls (c), in glucose- and fructose-grown* S. cerevisiae* YPH250. Results are shown as the mean ± SEM (*n* = 3–6). ^*∗*^Significantly different from respective values for glucose with *P* < 0.05.

**Figure 2:**
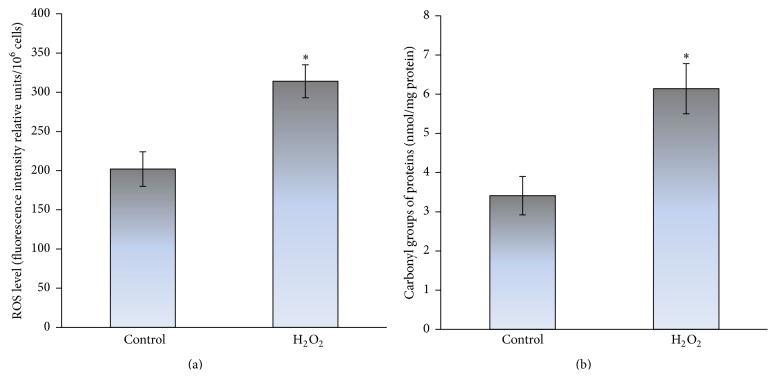
Effect of hydrogen peroxide on the level of oxidative stress markers: ROS (a) and protein carbonyls (b), in* S. cerevisiae* YPH250. Results are shown as the mean ± SEM (*n* = 3–7). ^*∗*^Significantly different from respective values for control cells (without H_2_O_2_) with *P* < 0.05.

Here, we used yeast cultures grown on glucose or fructose for 24 h, when the exponential phase merged slowly into the stationary phase of growth.  Figure 1 shows the influence of glucose and fructose on the intracellular level of the glycoxidation products. Fructose-grown as compared to glucose-grown cells demonstrated 2.5-fold higher metabolic rate (Figure 1(a)), which was consistent with our previous observations [11, 26]. It is well documented that overall metabolic rate, in particular carbohydrate metabolism, largely determines cellular redox balance [37, 38] and lifespan [39]. Significantly higher metabolic activity (Figure 1(a)) correlated with higher level of oxidative stress markers, ROS and protein carbonyls, in fructose-grown than glucose-grown cells (1.7- and 1.3-fold in Figures 1(b) and 1(c), resp.).  Figure 2 shows higher level of total ROS (1.6-fold) and carbonyl proteins (1.8-fold) in yeast treated with 10 mM H_2_O_2_ relative to the control cells (without H_2_O_2_). Therefore, the increased levels of ROS and carbonyl proteins in fructose-grown yeast similar to those in the cells exposed to hydrogen peroxide can be explained by the development of oxidative stress during fructose-supplemented growth.

**Figure 3:**
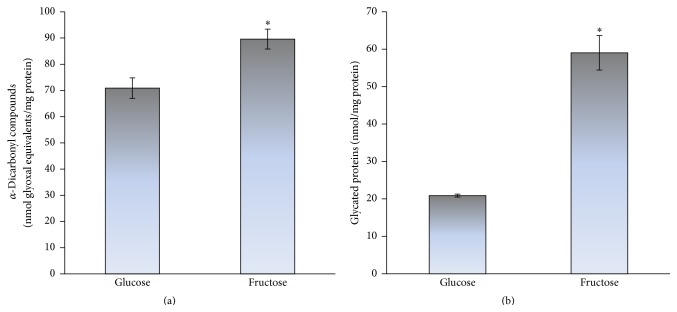
The level of carbonyl stress markers: *α*-dicarbonyl compounds (a) and glycated proteins (b), in glucose- and fructose-grown* S. cerevisiae* YPH250. Results are shown as the mean ± SEM (*n* = 4–6). ^*∗*^Significantly different from respective values for glucose with *P* < 0.05.

**Figure 4:**
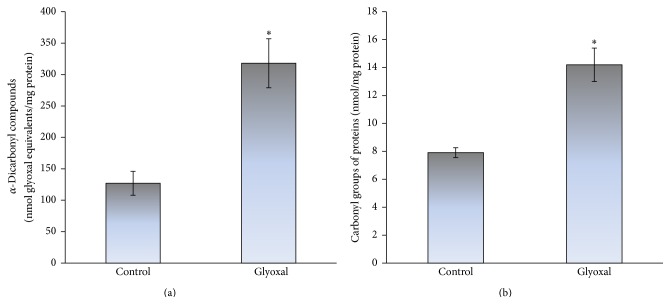
Effect of glyoxal on the level of carbonyl stress markers: *α*-dicarbonyl compounds (a) and protein carbonyls (b), in* S. cerevisiae* YPH250. Results are shown as the mean ± SEM (*n* = 3–7). ^*∗*^Significantly different from respective values for control cells (without glyoxal) with *P* < 0.05.

From the previous reports it is well known that the excessive fructose intake in animal [10, 12] and yeast models [11, 36] can also cause carbonyl stress. In addition, the increased protein carbonyls have been shown to accompany a number of physiological and pathological processes and were suggested to be a marker of aging [29, 40–44]. Therefore, next we examined the level of carbonyl stress markers in both the studied types of cells (glucose- and fructose-grown).  Figure 3 demonstrates higher level of *α*-dicarbonyl compounds (a) and glycated proteins (b) in fructose-grown cells than those in cells grown on glucose (1.3- and 2.8-fold, resp.). To compare the ability of fructose and highly reactive carbonyl compound glyoxal to cause carbonyl stress, next we measured the level of carbonyl stress markers in yeast stressed by 50 mM glyoxal. The contents of *α*-dicarbonyls (Figure 4(a)) and protein carbonyls (Figure 4(b)) were found to be significantly increased after yeast treatment with glyoxal (2.5- and 1.8-fold, resp.). Thus, fructose-supplemented yeast cultivation can cause both oxidative and carbonyl stresses.

**Figure 5:**
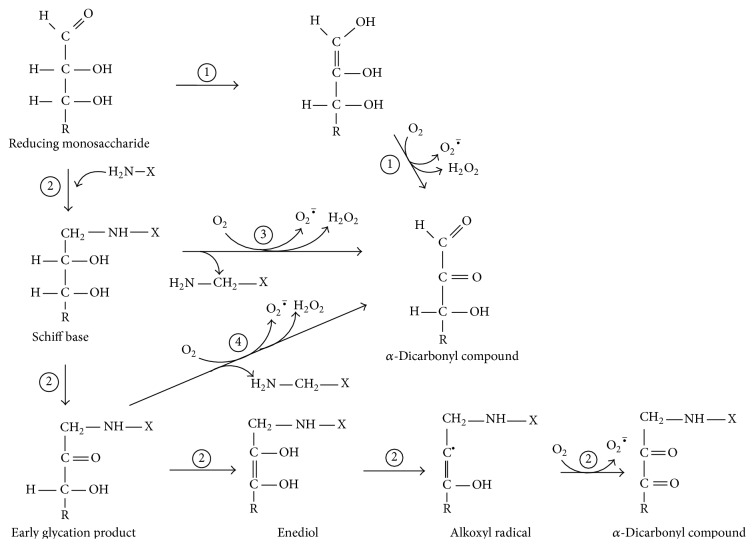
Involvement of reducing monosaccharides such as glucose and fructose in the glycoxidation reactions resulting in the production of ROS and RCS: (1) Wolff pathway; (2) glycation; (3) Namiki pathway; and (4) Hodge pathway.

Figure 5 demonstrates how glucose and fructose are involved in ROS/RCS formation and oxidative/carbonyl stress. Glycoxidation can be initiated by both the reducing monosaccharides and yields enediols and alkoxyl radicals. Alkoxyl radical readily reacts with molecular oxygen generating superoxide anion radical, which can be converted to hydrogen peroxide and then to hydroxyl radical. At the same time, glycoxidation results in the formation of *α*-dicarbonyls such as glyoxal and methylglyoxal. Experiments comparing fructose and glucose reactivity in nonenzymatic processes demonstrate higher reactivity of fructose [4, 11, 13, 14, 26, 36]. At first glance, it disagrees with the concept of organic chemistry that aldoses (e.g., glucose) due to a greater electrophilicity and accessibility of their carbonyl group are more reactive than respective ketoses (e.g., fructose). One explanation is that glucose is less reactive due to the formation of very stable ring structures in aqueous solutions which retards its reactivity. Fructose also forms cyclic structures but exists to a greater extent in the open-chain active form than glucose. Therefore, under conditions used in this study fructose compared to glucose is a more potent initiator of glycoxidation* in vivo* (Figures 1 and 3).

**Figure 6:**
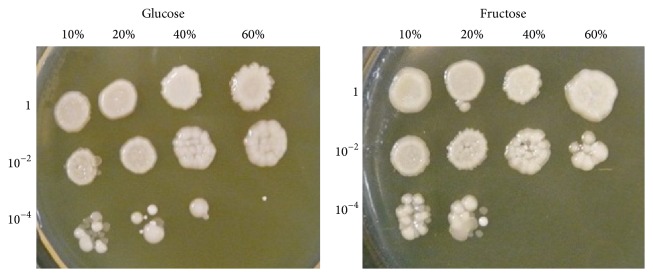
Cell viability assay of* S. cerevisiae* YPH250 cells after exposure to high concentrations of glucose and fructose. In all samples within each plate, cell concentration was equaled and successively diluted by a factor of 100 down to 10^−4^. Representative results from a set of three experiments are shown.

**Figure 7:**
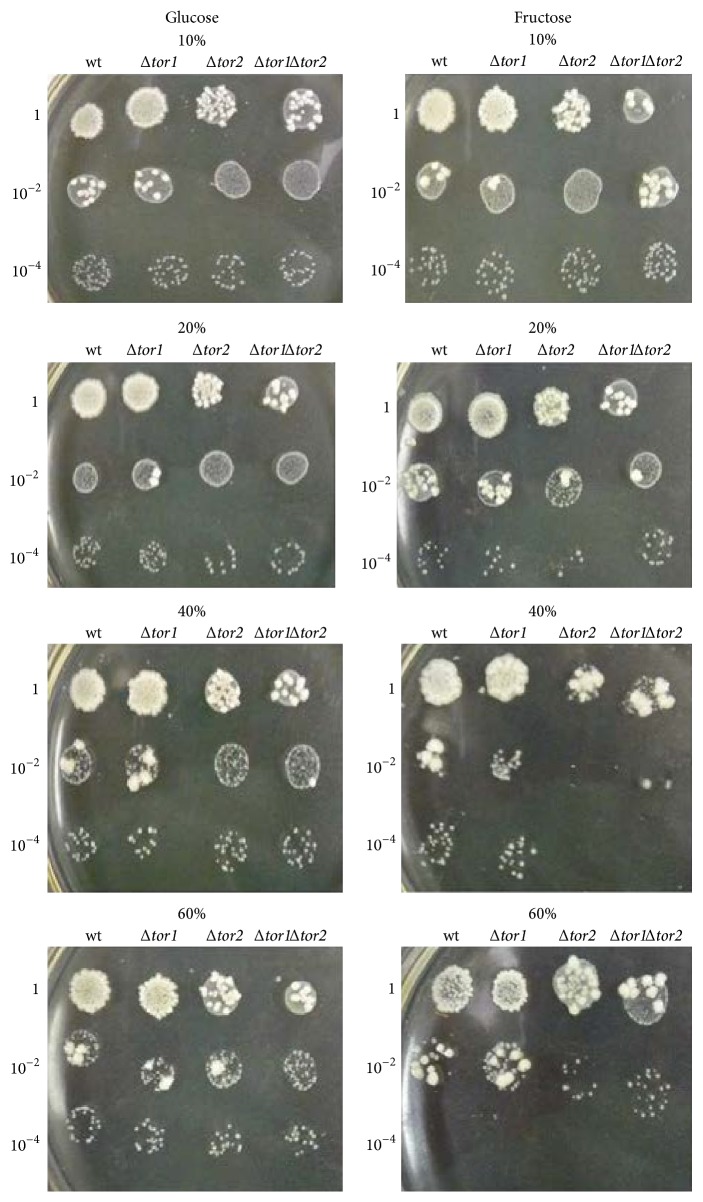
Cell viability assay of* S. cerevisiae* JK9-3da (wild type),* Δtor1*,* Δtor2*, and* Δtor1Δtor2* cells after exposure to high concentrations of glucose and fructose. In all samples within each plate, cell concentration was equaled and successively diluted by a factor of 100 down to 10^−4^. Representative results from a set of three experiments are shown.

The above mentioned is consistent with somewhat better survival of* S. cerevisiae* YPH250 after stress induced by 40% glucose than 40% fructose (Figure 6). Similar results were obtained when another wild type strain* S. cerevisiae* JK9-3da and 60% carbohydrates were used (Figure 7); but, in contrast to* S. cerevisiae* YPH250, no difference has been observed between glucose- and fructose-stressed* S. cerevisiae* JK9-3da in the case of 40% monosaccharides. Thus,* S. cerevisiae* JK9-3da seems to be more resistant to high concentrations of fructose than* S. cerevisiae* YPH250. It is interesting that derivatives of JK9-3da, single mutant* Δtor2* and double mutant* Δtor1Δtor2*, demonstrated lower survival under stress induced by 40% fructose than 40% glucose (Figure 7). Thus, some involvement of the TOR pathway can be supposed in fructose-induced stress in yeast. The previous work reported the decreased intracellular ROS levels at the inactivation of TOR signaling pathway by caloric restriction or the* tor1* gene deletion, while activation of the TOR signaling pathway in* S. cerevisiae* by high nutrient concentrations increased ROS levels [45]. In addition, recently it has been suggested that TOR inhibition suppressed formation of methylglyoxal and other deleterious RCS [46] produced during carbohydrate metabolism [47, 48]; however any experimental confirmation of these hypotheses had not yet been found.

**Figure 8:**
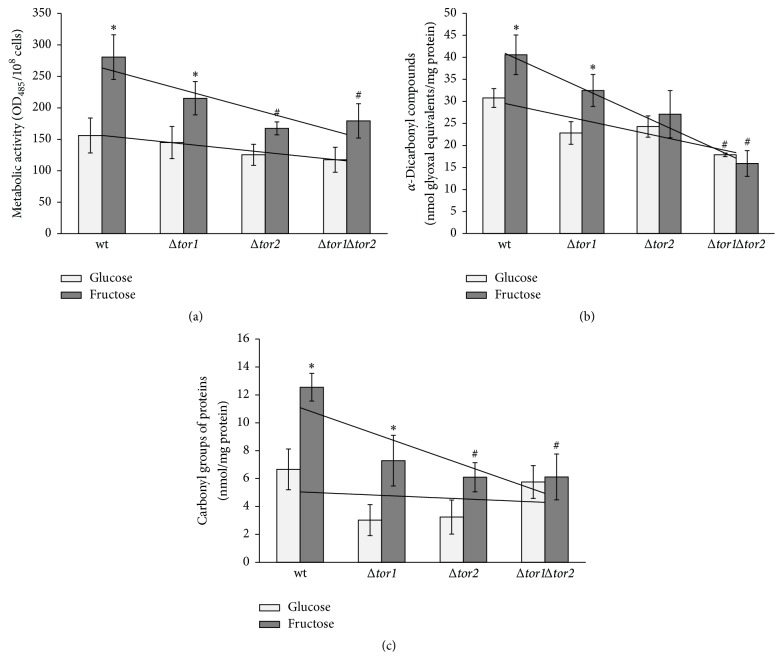
The metabolic rate (a) and level of carbonyl/oxidative stress markers: *α*-dicarbonyl compounds (b) and protein carbonyls (c), in glucose- and fructose-grown* S. cerevisiae* JK9-3da and its mutants* Δtor1*,* Δtor2,* and* Δtor1Δtor2*. Results are shown as the mean ± SEM (*n* = 3–6). ^*∗*^Significantly different from respective values for glucose and ^#^wild type with *P* < 0.05.

In order to examine the above-mentioned suggestion, the level of oxidative/carbonyl stress markers has been determined in the wild type JK9-3da and mutants defective in the* tor1* and* tor2* genes grown on glucose and fructose (Figure 8). In accordance with the suggestion that fructose versus glucose is a more potent inducer of oxidative/carbonyl stress and the data obtained on* S. cerevisiae* YPH250 (Figures 1 and 3), the metabolic rate (Figure 8(a)), level of *α*-dicarbonyls (Figure 8(b)), and protein carbonyls (Figure 8(c)) were found to be significantly higher in JK9-3da cells grown on fructose than those in the cells grown on glucose (1.8-, 1.3-, and 1.8-fold, resp.). The parameters in the three mutants grown on glucose demonstrated the values similar to those found in glucose-grown wild type cells. In the case of fructose-supplemented cultivation, all of the three parameters determined, metabolic activity, level of *α*-dicarbonyls, and protein carbonyl groups, were overall lower in the three mutants as compared to wild type (1.7-, 2.6-, and 1.9-fold, resp.). Therefore, the TOR gene deletions decreased the concentration of RCS in fructose-grown yeast. The latter corresponds well to the previous suggestion on suppressed RCS generation by TOR pathway inactivation [46].

## 4. Conclusion

The TOR signaling pathway first described in* S. cerevisiae* is highly conserved between yeast, animals, and plants. It received tremendous attention due to its importance in regulating cell growth, metabolism, longevity, and stress response. Our study confirms the previous findings on the higher reactivity of fructose versus glucose, more potent production of RCS/ROS, and development of oxidative/carbonyl stress in different fructose-supplemented models and extends them with the first report of the metabolic rate inhibition and lower generation of glycoxidation products in fructose-grown yeast lacking TOR proteins. Further research is needed to understand how TOR regulation may help prevent metabolic syndrome, diabetes complications, and other disturbances related to chronic consumption of diets high in fructose.
